# Serum Levels of Bone Morphogenetic Proteins 2 and 4 in Patients with Acute Myocardial Infarction

**DOI:** 10.3390/cells9102179

**Published:** 2020-09-27

**Authors:** Maria Kercheva, Anna M. Gusakova, Tamara R. Ryabova, Tatiana E. Suslova, Julia Kzhyshkowska, Vyacheslav V. Ryabov

**Affiliations:** 1 Siberian State Medical University, 634055 Tomsk, Russia; rvvt@cardio-tomsk.ru; 2 Cardiology Research Institute, Tomsk National Research Medical Center, Russian Academy of Sciences, 634012 Tomsk, Russia; mag_a@mail.ru (A.M.G.); rtrtom2@rambler.ru (T.R.R.); tes@cardio-tomsk.ru (T.E.S.); 3 National Research Tomsk State University, 634050 Tomsk, Russia; julia.kzhyshkowska@googlemail.com; 4 Institute of Transfusion Medicine and Immunology, University of Heidelberg, 1-3 Theodor-Kutzer Ufer, 68167 Mannheim, Germany

**Keywords:** myocardial infarction, BMP-2, BMP-4, macrophages, adverse left ventricular remodeling

## Abstract

Background: Bone morphogenetic proteins-2 and -4 (BMPs) have been implicated in left ventricular remodeling (LVR) processes such as an inflammation and fibrogenesis. We hypothesized that this knowledge could be translated into clinics. Methods: We studied the dynamics of serum levels of BMPs, its correlation with markers of LVR and with parameters of echocardiography in patients (*n* = 31) during the six-month follow-up period after myocardial infarction (MI). Results: Elevated serum levels of BMPs decreased by the six-month follow-up period. BMP-2 decreased from the first day after MI, and BMP-4 decreased from the Day 14. The elevated level of BMP-2 at Day 1 was associated with a lower level of troponin I, reperfusion time and better left ventricular ejection fraction (LV EF) at the six-month follow-up. Elevated serum level of BMP-4 at Day 1 was associated with a lower level of a soluble isoform of suppression of tumorigenicity 2 (sST2), age and reperfusion time. An elevated level of BMP-2 at the six-month follow-up was associated with higher levels of BMP-4, high-sensitivity C-reactive protein (hCRP) and sST2. High serum level of BMP-2 correlated with high levels of hCRP and matrix metalloproteinase (MMP)-9 on Day 7. High serum level of BMP-4 correlated with low levels of hCRP, MMP-9 at Day 3, sST2 at Day 1 and with decreased LV EF on Day 7. The findings of multivariate analysis support the involvement of BMP-2 in the development of post-infarction LVR. Conclusions: Our research translates experimental data about the BMPs in the development of adverse LVR into the clinic. Elevated serum levels of BMPs decreased by the end of the six-month period after MI. BMP-2 decreased from the first day and BMP-4 decreased from Day 14. BMP-2 and BMP-4 were associated with the development of LVR. Their correlations with markers of inflammation, degradation of the extracellular matrix, hemodynamic stress and markers of myocardial damage further support our hypothesis. Diagnostic and predictive values of these BMPs at the development of post-infarction LVR in vivo should be investigated further.

## 1. Introduction

Acute myocardial infarction (AMI) with subsequent development of chronic heart failure (CHF) remains one of the leading causes of disability in the world [1]. The frequency and progression of CHF in patients after MI remains high, despite modern methods of myocardial revascularization, the large number of established vascular centers and international standards for the management of this category of patients and multi-conservative therapy employed [2]. Chronic aseptic inflammation that occurs under hemodynamic stress and is characterized by the formation of fibrotic scar tissue is a basis for the development of post-infarction adverse left ventricular remodeling (LVR) and CHF progression [1,3]. In our previous research, we obtained data on cell–molecular predictors of the adverse LVR [4]. However, the mechanism of LVR development and the roles of the inflammatory response and fibrogenesis in heart remodeling remain poorly understood [5,6].

Bone morphogenetic proteins (BMPs), with their pleiotropic functions and roles in the regulation of inflammatory and reparative responses—in particular BMP-2 and BMP-4—are excellent subjects of scientific interest [6,7]. These proteins control the proliferation and cell differentiation, including myocardial cells, and are actively involved in gene expression [8,9]. It is believed that reactivation of the embryonic signaling pathways involved in the formation of heart tissue could improve functional survival rates of cardiomyocytes in patients after AMI [3,6]. However, the exact role of BMPs in fibrosis and inflammation has not yet been determined; the existing data are mainly experimental and often conflicting [3,10]. Currently, the experimental data indicate that the absence of the BMP-4 gene, which is associated with significant pathologies in heart formation [10], and additional administration of BMP-2, can reduce the infarction zone in the AMI model [3]. There are no clinical data about the serial assessment of the dynamics of serum levels of BMP-2 and BMP-4, their correlations with known markers of inflammation, degradation of the intercellular matrix and hemodynamic stress, with parameters of echocardiography in patients in the early and late post-infarction period. These results would provide us with an understanding of the role of BMPs in regeneration, inflammation and the development of postinfarction LVR in vivo. It allows implementing these data to further study BMPs in patients with AMI, as expected targets of effective antifibrotic therapy.

The purpose of our research was to translate experimental knowledge regarding the role of BMPs in post-infarction cardiac remodeling into results observed in real clinical settings. We studied the early and late dynamics of serum levels of BMP-2 and BMP-4 and its correlation with levels of high sensitive C-reactive protein (hCRP), *N*-terminal pro-brain natriuretic peptide (NT-proBNP), matrix metalloproteinase-9 (MMP-9), a soluble isoform of suppression of tumorigenicity 2 (sST2) and also with parameters of echocardiography at Days 1, 3, 7 and 14 and at 6 months in patients with ST-segment elevation MI (STEMI).

## 2. Materials and Methods

### 2.1. Clinical Data Analysis

The study involved 31 patients with acute primary anterior STEMI who were admitted to the emergency cardiology department during the first 24 h from the onset of MI [4]. Percutaneous coronary intervention (PCI) was performed on the first day of hospitalization. The identification number of the study in the international database ClinicalTrials.gov is NCT02562651. Exclusion criteria included the following: more than 75 years of age, severe comorbidities, Killip Class IV associated with index MI, non-Q-wave MI, heart rate < 40 beats per minute, chronic atrial fibrillation, decompensated HF (New York Heart Association (NYHA) Class III–IV), severe valvular heart disease or poor quality of imaging data. All procedures performed in studies involving human participants were in accordance with the ethical standards of the institutional and/or national research committee and with the 1964 Helsinki declaration and its later amendments or comparable ethical standards and Good Clinical Practice Guidelines in the Russian Federation approved by the Ministry of Health of the Russian Federation on 1 April 2016 and was endorsed by the local ethics committee. Informed consent was obtained from all individual participants included in the study.

Diagnostics and treatment were conducted in accordance with European recommendations for the diagnosis and treatment of patients with acute STEMI [2]. STEMI was diagnosed based on the third universal definition of MI [2]. Echocardiography was performed in all patients at Days 3 (T2), 7 (T3) and 14 (T4) and six months (T5) after STEMI onset (Vivid E9, GE Healthcare, Boston, MA, USA) [4,11,12]. The obtained images were stored on CD-ROM drives and then analyzed using GE EchoPAC software (GE, Healthcare, EchoPac 113, Boston, MA, USA). Left ventricle (LV) end-diastolic volume (EDV) and end-systolic volume (ESV) and the LV ejection fraction (EF) were estimated using the biplane Simpson’s method [13]. We assessed the development of adverse LVR in the early (by the 14th day) and late (by six months) periods after AMI according to the enlargement of EDV and/or ESV LV more than 20% from the baseline data [4].

### 2.2. Assays

We analyzed serum samples on Day 1 (T1) and at the same terms, as echocardiography data. To obtain serum, blood was incubated at room temperature for 20–30 min for a clot to form and then centrifuged for 15 min at room temperature at a speed of 3000 rpm. To obtain plasma, blood was centrifuged immediately. We determined serum level of hCRP using a CRP-hs kit (BIOMERICA, Irvine, CA, USA); NT-proBNP was determined using an NTproBNP test system (Biomedica, Bratislava, Slovakia); MMP-9 were measured using the human MMP-9 kits and R&D Systems, USA; sST2 was determined using the ThePresage^®^ ST2 Assay^®^ (Critical Diagnostics, San Diego, CA, USA); BMP-2 was measured using the human BMP-2 ELISA kit (RayBioTech, Norcross, GA, USA); BMP-4 was measured using the human BMP-4 ELISA kit (RayBioTech, Norcross, GA, USA). We measured serum levels of troponin I using AccessAccuTnI the Access 2 immunoassay system, Beckman Coulter, Brea, CA, USA. Reference values of serum levels of BMP-2 (17.1 ± 0.6 pg/mL) and BMP-4 (173.2 ± 45.4 pg/mL) were the same as the healthy controls [14,15].

### 2.3. Data Analysis and Statistics

Data analysis was carried out using STATISTICA 10 software. The results are presented in the form of M ± SD for normal distribution of data or the median (Me) and lower and upper quartiles (Q1; Q3) for abnormal data distribution. The critical significance level is *p* < 0.05. We used the Student *t-*test for data analysis in case of the normal distribution; the Friedman test, the Mann–Whitney U-test and the Kruskal–Wallis test for abnormal distribution. The data obtained by the researchers were compared using correlation analysis (Spearman’s rank correlation coefficient for normal distribution, Pearson’s correlation coefficient for abnormal distribution). The correlation coefficient value r = 0.4–0.7 indicated relatively strong correlation, r > 0.7 indicated strong correlation [16]. We used a multivariable logistic regression model to select the predictive variables of the development of adverse LVR at the early and late periods of MI. The following variables were included in the multivariable logistic regression model: reperfusion time, complete revascularization and troponin I as standard markers of adverse LVR and also hCRP, MMP-9 and sST2, as parameters with the linear associations with BMP-2 and -4 and BMP-2 and BMP-4 levels, respectively.

## 3. Results

### 3.1. Baseline Characteristics

The study involved 31 patients (mean age 58.3 ± 9.8 years) with STEMI who were admitted to the emergency cardiology department between 1 March 2014 and 1 March 2015 [4]. The medical history and vital signs of patients are presented in Table 1 and Table 2.

**Figure 1:**
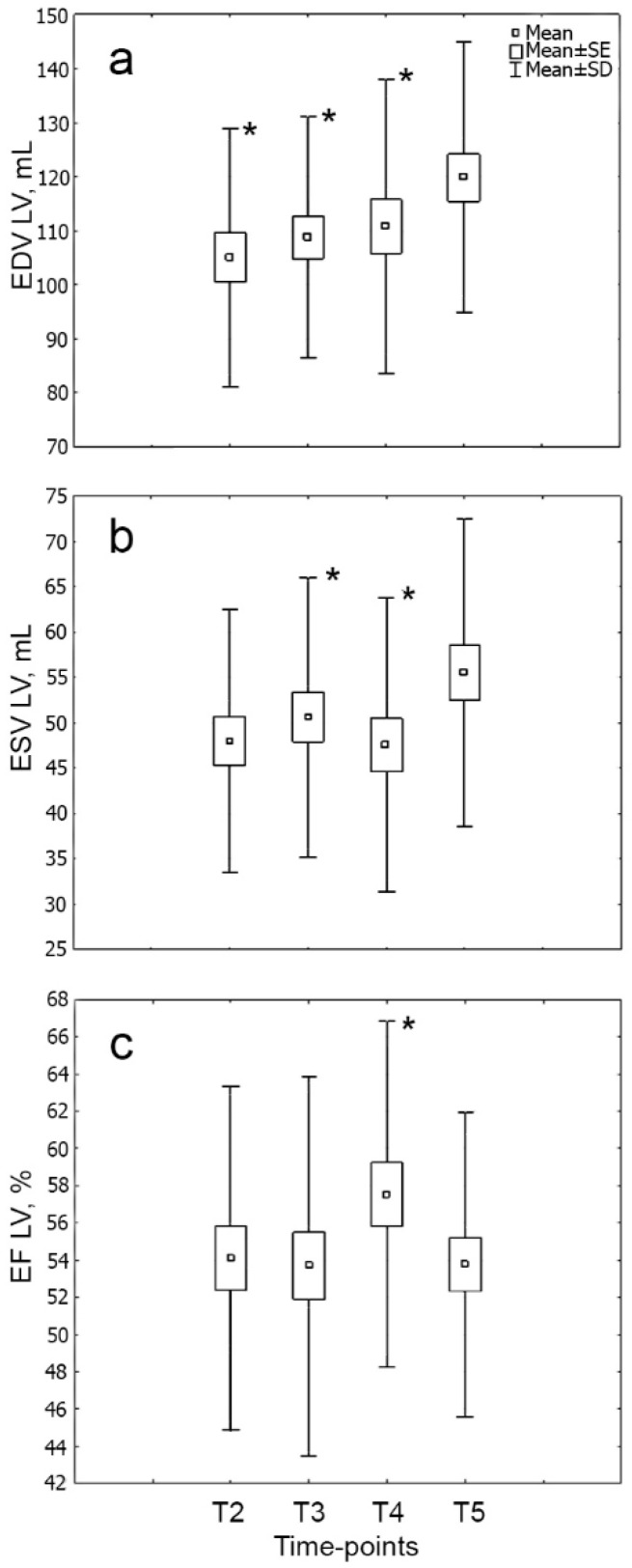
Dynamics of the main echocardiographic parameters ((**a**) EDV, (**b**) ESV, (**c**) EF) of LV in the six-month period in patients with STEMI (*n* = 31). Note: * *p* < 0.05: significant difference between time point T5 and other time points. EDV—end-diastolic volume, ESV—end-systolic volume, LV—left ventricle, EF—ejection fraction; T2—3rd day; T3—7th day; T4—14th day; T5—6 months after MI [4].

The dynamics of echocardiography parameters are shown in Figure 1.

The most common complication in the early period of MI was cardiac arrhythmias (29%) and acute HF (Killip class ≥ II) was observed in 9% of cases. There was no single cause of a fatal outcome during the entire follow-up period; recurrent MI developed in 3% of cases. After six months, FC ≥ III stable angina was found in 3% of cases and NYHA FC > I CHF was recorded in 31% of cases [4].

We revealed associations between the serum levels of BMP-4 at the admission and with a history of musculoskeletal disorders (r = 0.6, *p* < 0.05), with the patient’s age (r = −0.6, *p* < 0.05). The serum level of BMP-2 was associated with reperfusion time (r = −0.7, *p* < 0.05).

### 3.2. Dynamics of BMP-2

**Figure 2:**
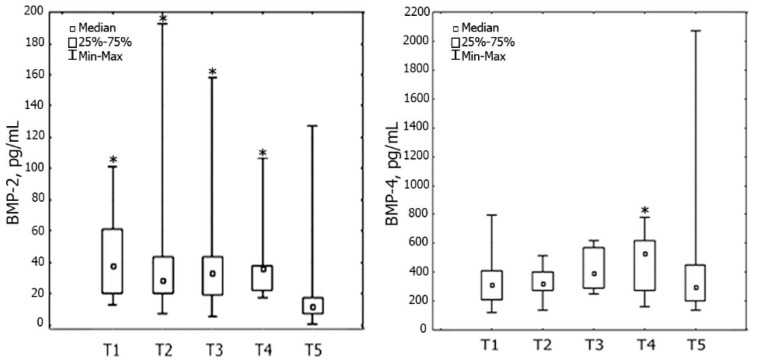
Dynamics of serum levels of BMP-2 and BMP-4 (*n* = 31) in the six-month period in patients with STEMI. Note: * *p* < 0.05: significant difference between time point T5 and other time points. BMP—bone morphogenetic protein; T1—1st day; T2—3rd day; T3—7th day; T4—14th day; T5—6 months after MI.

Serum levels of BMP-2 and BMP-4 decreased by the end of the six-month period after AMI; however, the dynamics of this decrease were different (Figure 2).

During the whole follow-up period, the mean value of serum levels of BMP-4 and BMP-2 remained higher than the reference values.

Upon hospital admission, the serum level of BMP-2 was higher than the reference value in 75% of patients (*n* = 23). These patients had lower level of troponin I 30.8 (0.5–80.9) vs. 88 (46–144) ng/mL (*p* = 0.03) at time-point T1, and reperfusion time was also less (3.8 (2.08–8.4) vs. 10.5 (7–17) h) than in other patients (*p* = 0.02). However, echocardiography parameters were better in these patients by time-point T5: ESV LV 53 (30–82) vs. 70 (55–87) mL (*p* = 0.03) and EF LV 54 (42–65) vs. 45 (42–48)% (*p* = 0.01). Other clinical and medical history data did not differ between these groups.

The serum level of BMP-2 was increased in 25% of patients (*n* = 8) by time-point T5. These patients had lower ESV LV 37 (33–42) vs. 58 (44–72) mL at time-point T2 (*p* = 0.04). However, the serum levels of BMP-4 at time-point T5 were higher than in other patients 1111 (585–2069) vs. 231 (135–344) pg/mL (*p* = 0.02), such as the levels of hCRP 6.4 (3.7–8.4) vs. 1.7 (0.6–3.1) mg/L (*p* = 0.01); sST2 38 (31–47) vs. 27 (24–32) ng/mL (*p* = 0.01) and MMP-9 554 (125–2066) vs. 125 (17.9–269) ng/mL (*p* = 0.02).

The serum level of BMP-2 decreased on the first day after AMI and continued throughout the entire six-month follow-up: 40 (12; 101) at time-point T1; 39 (7; 192) at time-point T2, 42 (5; 158) at time-point T3, 37 (17; 107) at time-point T4; 20 (0.7; 127) pg/mL at time point-T5 (*p* = 0.001).

### 3.3. Dynamics of BMP-4

The serum level of BMP-4 was higher than the reference values in 70% of patients (*n* = 21) upon hospital admission. These patients had a lower level of sST2 at time-point T1 relative to the other patients (64 (27–201) vs. 159 (54–314) ng/mL (*p* = 0.03)), their reperfusion time was less (5.1 (2.08–17) vs. 5.8 (4.3–7) h) and they were younger (57 (44–69) vs. 65 (57–70) years) than in other (*p* = 0.04). Other clinical and medical history data did not differ between groups. Within the period from the 14th day after AMI to the end of the six-month follow-up, the serum level of BMP-4 decreased by 37%: from 464 (161; 776) to 436 (135; 2069) pg/mL (*p* = 0.02). There were no significant changes in the serum level of BMP-4 during the first 14 days after AMI: 336 (120; 794) at time-point T1; 335 (134; 157) at time-point T2; 422 (253; 621) pg/mL at time-point T3.

An increased serum level of BMP-4 was found in 75% of patients (*n* = 8) by time-point T5. Clinical and medical history data did not differ between groups.

### 3.4. Correlations between BMP-2 and BMP-4 and Serum Levels of hCRP, MMP-9, sST2 and NT-proBNP

**Figure 3:**
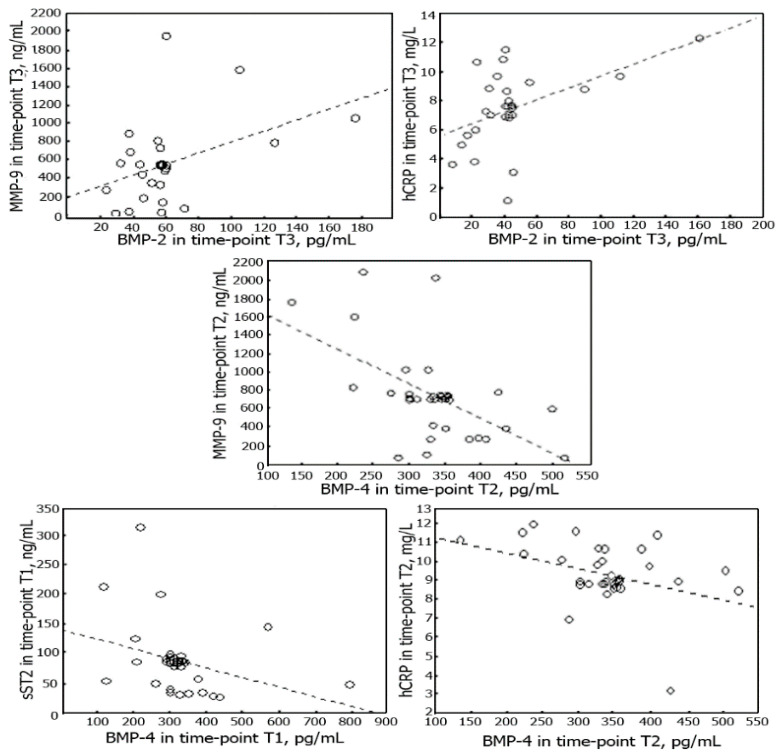
Scatterplots of BMP-2 and BMP-4 and sST2, MMP-9 and hCRP during the 6 months after MI. Spearman’s rank-order correlation: between BMP-2 and MMP-9 (r = 0.5, *p* = 0.03) and between BMP-2 and hCRP (r = 0.5, *p* = 0.03) at time-point T3; between BMP-4 and hCRP (r = −0.6, *p* = 0.04) at time-point T2 and between BMP-4 and MMP-9 (r = −0.5, *p* = 0.03) at time-point T2; between BMP-4 and sST2 at time-point T1 (r = −0.5, *p* = 0.03).

Serum level of BMP-2 correlated with the serum levels of hCRP and MMP-9 measured at time-point T3. The serum level of BMP-4 correlated with the serum level of sST2 measured at time-point T1; at time-point T2 it correlated with the levels of MMP-9 and hCRP. There were no correlations between the serum levels of NT-proBNP and BMPs. All correlations are shown in Figure 3. The correlation was also observed between serum levels of BMP-2 and BMP-4 at time-point T5 (r = 0.6, *p* = 0.03).

### 3.5. Correlations between BMP-2 and BMP-4 and Adverse LVR in Both Early and Late Periods of AMI

In our group, 14 patients had adverse LVR at the late period of MI and 17 had LVR in the early period of MI. Correlation analysis revealed only one association between the levels of BMP and echocardiography parameters; it was found between the BMP-4 level and LV EF at time-point T3 (r = −0.5, *p* = 0.04). We found no associations between the levels of BMP-2 and BMP-4 and the development of adverse LVR in the late period of MI. However, in the multivariate analysis model of the development of adverse LVR in the early period of MI, we included the following parameters: reperfusion time, complete revascularization, level of troponin I, sST2, hCRP, MMP-9 and BMP-2 and BMP-4, respectively. Associations were found with the level of troponin I (*p* = 0.004), sST2 (*p* = 0.02), reperfusion time (*p* = 0.01) and with the level of BMP-2 (*p* = 0.03) (Table 3).

## 4. Discussion

One of the most controversial and unresolved problems in modern emergency cardiology is the assessment of the role of inflammation in the development and progression of adverse LVR and following CHF [17,18,19]. It is known that acute ischemic myocardial damage and death of cardiomyocytes trigger inflammatory response [20]. However, timely suppression of inflammatory signals in the infarction zone creates optimal conditions for scarring and prevents the development of adverse LVR [20]. Recently, scientists have actively studied innate immunity cells, macrophages, which are found in both healthy and damaged tissues, including ischemic myocardium [20,21,22]. Two subpopulations of macrophages are currently known: classically activated (M1) macrophages and alternatively activated (M2) [20]. The data on the participation of M1 macrophages in inflammation processes have been obtained, whereas M2 macrophages remain poorly studied. Currently, mainly experimental data are used in studies since the results of clinical trials are few and contradictory [20]. Bone morphogenetic proteins are actively secreted including by M2 macrophages and coordinate inflammatory processes in cells of various types, from immune cells to endothelial and connective tissue cells [3]. They could also influence deviations of spatial and temporal regulation of inflammation after MI [3]. The obtained data for the dynamics of these proteins and their role in the development of adverse LVR will likely help us to affect the plasticity of macrophages in the infarction zone and hence to make appropriate changes in the course of regeneration and cardiac remodeling during and after MI. To date, it has been shown experimentally that BMPs cause changes in the expression of cardiac proteins [6], which affect the growth, aging and differentiation of cells [23] and actively suppress fibrosis after MI. This influence of BMPs could reduce the degree of damage to myocardial tissue and stimulating the regeneration of functional myocardial tissue in the infarction zone [1]. It is also known that BMPs are involved in the differentiation and formation of various progenitor cells during embryogenesis, such as cardiac [6], skeletal and smooth muscle cells [23,24]. This likely explains the presence of correlations between the values of BMP-4 with the history of musculoskeletal disorders pathology and with patients’ age in our sample. Our data show that serum levels of BMP-2 and BMP-4 were elevated at the admission and the entire follow-up period, which could be due to the possibility of BMP-2 increasing the expression of other BMPs [25]. However, BMP-2 begins to actively decrease on the first day after onset, while the serum level of BMP-4 decreased only on Day 14 after MI. This can indirectly indicate that BMP-2 is more actively involved in the early post-infarction myocardial remodeling. This involvement of BMP-2 in the development of adverse LVR at the early postinfarction period along with standard markers of LVR is confirmed by the data of multiple regression models. According to the previous data, the role of BMP-2 in post-infarction regeneration is more experimentally investigated in comparison with other BMPs [3,10]. The correlation between the increased level of BMP-2 and high levels of MMP-9 and hCRP observed on Days 1 and 7 after MI, as well as at the six-month follow-up, most likely indicates that BMP-2 is an active driver of the myocardium inflammation under ischemia conditions. The association of a high level of BMP-2 at Day 1 with better contractility of LV at six-month follow-up, as well as the association between a high level of BMP-2 at six months period with an increased level of MMP-9, sST2 and hCRP could explain the involvement of BMP-2 in the processes of early LVR and reflect the standard stream of inflammation after MI [20,25,26].

On the contrary, we found that the decreased serum level of sST2 on the first day after MI and serum levels of hCRP and MMP-9 on the third day after MI correlate with high serum BMP-4 levels, which may indicate that BMP-4 exhibits anti-inflammatory activity. In addition, BMP-2 is known to induce chemotaxis of monocytes and disrupt their differentiation into anti-inflammatory M2 macrophages [27]. Perhaps, this can explain the absence of significant dynamics of serum levels of BMP-4 with anti-inflammatory activity in the early period of AMI in our study. However, under experimental conditions, the researchers previously noted an increase in the expression of the BMP-4 protein precursor 24 h after onset of ischemia, which then gradually decreased [28,29,30,31]. Thus, the obtained data confirm a controversial and multifaceted role of BMPs in the development of adverse LVR in patients after AMI. Initially, BMPs signaling can play a favorable role; it involves endothelial cells to attract infiltrating leukocytes into the tissue and increases the value of the profibrotic signal [3]. However, the long-term impact of a damaging factor, inflammation, can cause uncontrolled activation of leukocytes and facilitate their transformation into tissue healing antagonists, thereby helping to enhance tissue fibrosis. These new clinical results about BMPs and their dynamics help translate experimental data into the clinical context. The involvement of BMP-2 and BMP-4 in processes of inflammation and regeneration, as in the development of LVR, support the importance of the further study of BMPs in clinics and could be the next step to creating effective antifibrotic targets.

## 5. Conclusions

Our research translates experimental data about the BMPs in the development of adverse LVR into the clinic. Elevated serum levels of BMPs decreased by the end of the six-month period after MI. BMP-2 decreased from the first day, and BMP-4 decreased from Day 14. BMP-2 and BMP-4 were associated with the development of LVR. Their correlations with markers of inflammation, degradation of the extracellular matrix, hemodynamic stress and markers of myocardial damage confirmed this. Diagnostic and predictive values of these BMPs at the development of post-infarction LVR in vivo should be investigated further.

## 6. Limitations

This study was conducted as a single-center trial in a small sample with strict inclusion criteria. Despite the fact that strict criteria were used, the conditions of real clinical practice may not reflect the experimental data in full volume due to the influence of a large number of external factors on the course of the disease. For these reasons, further studies are required.

## Figures and Tables

**Table 1:** Medical history and vital signs of patients with STEMI during the period of hospitalization (*n* = 31) [4].

At the Admission	
**Parameters**	
Male, *n* (%)	21 (67)
Smoking history, *n* (%)	15 (58)
BMI (kg/m^2^)	28 ± 5.1
Hypertension history, *n* (%)	21 (67)
Diabetes mellitus, *n* (%)	8 (25)
History of musculoskeletal disorders, such as osteoarthritis and osteochondrosis, *n* (%)	5 (16)
Dyslipidemia, *n* (%)	23 (74)
**Killip class, *n* (%)**	
1	28 (91)
2	1 (3)
3	2 (6)
**At the discharge**	
Combined endpoint (death, recurrent MI, angina pectoris FC ≥ III, HF NYHA class > I), *n* (%)	7 (22)
Recurrent MI, *n* (%)	1 (3)
HF NYHA class > I, *n* (%)	5 (16)
Angina pectoris FC ≥ III, *n* (%)	1 (3)

Notes: BMI—body mass index; FC—functional class; HF—heart failure; MI—myocardial infarction; NYHA—New York Heart Association; STEMI—myocardial infarction with ST-segment elevation.

**Table 2:** Examination data for patients involved in the study (*n* = 31) [4].

Parameters	
Extent of CAD, *n* (%)	
1-vessel CAD, *n* (%)	22 (71)
2-vessel CAD, *n* (%)	7 (23)
3-vessel CAD, *n* (%)	2 (6)
Reperfusion time (hours)	4.8 ± 3.3
First 3 h, *n* (%)	9 (29)
Thrombolysis + PCI, *n* (%)	17 (54)
Primary PCI, *n* (%)	14 (45)
Delayed PCI, *n* (%)	14 (45)
Complete revascularization, *n* (%)	20 (64)
LV EDV (Day 3), mL	107 ± 21.9
ΔLV EDV (Day 3, after 6 months),%	13.0 ± 20.0
LV ESV (Day 3), mL	49 ± 15.1
ΔLV ESV (Day 3, after 6 months),%	19.6 ± 40
LV EF (Day 3),%	54.2 ± 9.2
ΔLV EF (Day 3, after 6 months),%	2.9 ± 7.6

Note: CAD—coronary artery disease; PCI—percutaneous coronary intervention; EDV—end diastolic volume; ESV—end systolic volume; EF—ejection fraction; LV—left ventricular.

**Table 3:** Summary of multiple linear regression of reperfusion time, complete revascularization, level of troponin I, sST2, hCRP, MMP-9, BMP-2 and BMP-4 and diagnosis on adverse LVR.

Variable	β (Standard Deviation)	T	*p* Value
Complete revascularization, *n*	0.7	12.3	0.06
Reperfusion time, h	0.5	22.9	0.02
BMP-4, pg/mL	0.7	9.4	0.06
BMP-2, pg/mL	2.3	20.4	0.03
Troponin I, ng/mL	1.0	15.8	0.04
hCRP, mg/L	0.2	6.5	0.09
sST2, ng/L	−2.5	−22.2	0.02
MMP-9, ng/mL	0.6	10.7	0.06

BMP—bone morphogenetic protein; hCRP—high-sensitivity C-reactive protein; LVR—left ventricular remodeling; MMP—matrix metalloproteinases; sST2—soluble ST2; T2—3d day after myocardial infarction.

## Abbreviations

AMI	Acute myocardial infarction
BMI	Body mass index
BMP	Bone morphogenetic protein
CAD	Coronary artery disease
CHF	Chronic heart failure
ECG	Electrocardiogram
EDV	End-diastolic volume
EF	Ejection fraction
ESV	End-systolic volume
FC	Functional class
hCRP	High-sensitivity C-reactive protein
HF	Heart failure
LV	Left ventricular
LVR	Left ventricular remodeling
MMP	Matrix metalloproteinases
NT-proBNP	N-terminal pro-brain natriuretic peptide
PCI	Percutaneous coronary intervention
RFBR	Russian Foundation for Basic Research
sST2	Soluble isoform of suppression of tumorigenicity 2
STEMI	ST-segment elevation myocardial infarction
